# Plantar Pressure and Contact Area Measurement of Foot Abnormalities in Stroke Rehabilitation

**DOI:** 10.3390/brainsci11091213

**Published:** 2021-09-14

**Authors:** Ligia Rusu, Elvira Paun, Mihnea Ion Marin, Jude Hemanth, Mihai Robert Rusu, Mirela Lucia Calina, Manuela Violeta Bacanoiu, Mircea Danoiu, Daniel Danciulescu

**Affiliations:** 1 Sport Medicine and Physiotherapy Department, University of Craiova, 200585 Craiova, Romania; ligiarusu@hotmail.com (L.R.); elvira_paun@yahoo.com (E.P.); robert.rusu@edu.ucv.ro (M.R.R.); mirela.calina@edu.ucv.ro (M.L.C.); manuela.bacanoiu@edu.ucv.ro (M.V.B.); mircea.danoiu@edu.ucv.ro (M.D.); daniel.danciulescu@edu.ucv.ro (D.D.); 2 Faculty of Mechanics, University of Craiova, 200585 Craiova, Romania; mihnea_marin@yahoo.com; 3 Department of ECE, Karunya Institute of Technology and Sciences, Coimbatore 641114, India

**Keywords:** stroke, biomechanic evaluation, neurorehabilitation, plantar pressure, contact area, gait

## Abstract

Background: Evaluation of plantar pressure in stroke patients is a parameter that could be used for monitoring and comparing how the timing of starting a rehabilitation program effects patient improvement. Methods: We performed the following clinical and functional evaluations: initial moment (T1), intermediate (T2), and final evaluation at one year (T3). At T1 we studied 100 stroke patients in two groups, A and B (each 50 patients). The first group, A, started rehabilitation in the first three months after having a stroke, and group B started after three months from the time of stroke. Due to the impediments observed during rehabilitation, we made biomechanic evaluation for two lots, I and II (each 25 patients). Assessment of the patient was carried out by clinical (neurologic examination), functional (using the Tinetti Functional Gait Assessment Test for classifying the gait), and biomechanical evaluation (maximal plantar pressure (Pmax), contact area (CA), and pressure distribution (COP)). Results: The Tinetti scale for gait had the following scores: for group A, from 1.34 at the initial moment (T1) to 10.64 at final evaluation (T3), and for group B, 3.08 at initial moment (T1) to 9 at final evaluation (T3). Distribution of COP in the left hemiparesis was uneven at T1 but evolved after rehabilitation. The right hemiparesis had uniform COP distribution even at T1, explained by motor dominance on the right side. CA and Pmax for lot I increased more than 100%, meaning that there is a possibility for favorable improvement if the patients start the rehabilitation program in the first three months after stroke. For lot II, increases of the parameters were less than lot I. Discussions: The recovery potential is higher for patients with right hemiparesis. Biomechanic evaluation showed diversity regarding compensatory mechanisms for the paretic and nonparetic lower limb. Conclusions: CA and Pmax are relevant assessments for evaluating the effects on timing of starting a rehabilitation program after a stroke.

## 1. Introduction

Stroke has an important social impact and involves high costs regarding rehabilitation. The rehabilitation protocol after stroke has a lot of gaps regarding the use of specific measurements that could help to design the goals of rehabilitation. This study is the result of a review of the literature regarding rehabilitation after stroke.

In the first three months after a stroke, the muscle mass decreases by 1.88% to 3.74% (muscle atrophy), when comparing paretic and nonparetic segments [1]. Sometimes the atrophy is higher and tends to be at 24% in first six months after stroke [1,2]. Regarding this problem, it is important to discover the optimal time for starting rehabilitation after a stroke, and evaluate how patient improvement correlates to this timing.

Currently, strokes are one of the most important factors that cause disability and functional limitations, in patients, as well as affect daily activities. Additionally, survivors have a high fall risk, which can result in other medical consequences [3].

Mackintosh et al., in their research regarding how to monitor balance disorders, stability and falls, found that 36% of stroke patients have balance disorders and falls, whereas 24% of healthy people of the same age have balance disorders and suffer from falls [4].

All specific phenomena of cortical origin are effects of neuromotor control, and also involve effects of the morphofunction of the foot. Morphofunctional changes of the foot mean also affect the proprioceptive system [5]. For this reason, neuromotor rehabilitation starts with restoring the proprioceptive system function, increasing the chances of having improved carrying of afferent information and therefore reducing fall risk [6,7].

The complex approach of healing stroke patients needs not only clinical evaluation but also a large number of evaluation methods that allow us to make predictions regarding the evolution of functional rehabilitation. In this scope, biomechanic evaluation could contribute to understanding the morphofunctional changes of the foot in stroke patients, starting from plantar pressure assessment to contact area assessment of drop foot.

Studying the literature showed us that plantar pressure is a specific element which is in connection with foot alignment and static foot, but also in relation with weight distribution [8,9]. Plantar pressure, contact area (CA) and center of pressure (COP) are changed when morphofunctional foot disorders are developed [8], and this could give information about the foot features of stroke patients.

At the same time, there are a lot of questions about the kinetic changes that take place after a stroke, and in this context measuring the plantar pressure in correlation with other gait parameters, such as trajectories, seems to be a challenge for clinical assessment and physical therapy intervention [10]. 

Spasticity on the plantar flexors generates drop foot, and COP seems to have an anterior movement and generates plantar flexors activity and increases the foot inversion [11]. The relationship between evolution of COP and morphofunctional drop foot changes have been studied by many authors that report changes of the subtalar joint angle and foot axis [12].

Analysis of the literature highlights the lack of the non-invasive studies regarding how it is possible to evaluate the functional status of morphofunctional drop foot changes. We only found clinical studies about functional evaluation or gait analysis.

With this study, we are attempting to address the following problems. Analysis of the effect of early rehabilitation program and how we to quantify the results is needed, because the evolution of the spasticity [13] is uncertain. The second problem is the impact of the adaptive mechanisms that are developed for motor compensatory strategy. 

The aim of this study is to present biomechanic parameters that define the drop foot in stroke, and give guidance on how soon after having a stroke rehabilitation should be started. 

For our study, we chose to analyze the following parameters: maximal pressure for plantar regions, contact area, pressure distribution during gait, and center of pressure.

These parameters define the movement patterns which are correlated with functional changes of the foot, and also with the side lesion on the right or left side.

## 2. Materials and Methods

### 2.1. Study Design

#### 2.1.1. Subjects

The research consists in three moments of clinical and functional evaluation: T1 (initial moment), T2 (intermediate 3 weeks after stroke, 3 months, 6 months), T3 (final evaluation 1 year). We proposed three intermediate moments because we needed to monitor the entire rehabilitation program, but biomechanic evaluation was made at 6 months (moment T2) and 1 year after (moment T3) only for lot I and lot II (the lots are described below).

At the first moment (T1) we included in the study 100 stroke patients at average age of 60 years old, which were enrolled in the study between 2019–2020. We proposed two groups, A and B, depending on the moment of the start of the rehabilitation program with physical therapy.

First group A was 50 stroke patients that the rehabiliation program less than 3 months after stroke. Group A was 58% male patients and 42% female patients.

Group B was 50 stroke patients that started the rehabilitation program after 3 months from the stroke. Group A was 56% male patients and 44% female patients.

Due to the impediments observed during rehabilitation program, such as inconsistency in participation in the rehabilitation program (because of location and/or health status), we considered that was more relevant to make the biomechanic evaluation for a small number of patients, even if the clinical and functional evaluations show a better evolution of group A than group B. Therefore, we decided to organize 2 lots for biomechanic evaluation, lot I and lot II, for evaluation at 6 months (T2) and 1 year after stroke (T3).

Lot I included 25 patients (19 patients with severe gait disorders, score 0, and 6 patients with less obvious gait disorders, score 2). This lot came from group A and included 16 males (64%), mean body mass 86.50 (±5.82) kg, mean height 178.31 (±4.92) cm, and 9 females (36%), mean body mass 67.78 (±4.79) kg, mean height 161 (±4.06) cm.

Lot II included 25 patients (3 patients with severe gait disorders, score 0, and 12 patients with medium gait disorders, score2). This lot came from group B and included 14 males (56%), mean body mass 88.50 (±4.55) kg, mean height 180.14 (±3.80) cm, and 11 females (44%), mean body mass 69.23 (±4.38) kg, mean height 161.91 (±3.70) cm.

For classifying the gait, we used the Functional Gait Assessment Test (a modification of 8 Item Dynamic Gait Index) which includes 10 items, each item is scored from 0 to 3.

- 0 is severe impairment;
- 1 is moderate impairment;
- 2 is mild impairment;
- 3 is normal locomotion.

The average age was 58.48 years (41–76 years old) for lot I and 61.56 years (45–72 years old) for lot II.

We organized the groups and then the lots according to the following criteria:

1. Inclusion criteria:
  (1) Iskemic stroke;
  (2) Stability of neurologic lesion and vital function;
  (3) Minimum of 2 disability levels—the levels of disabilities take in consideration gait disorders and dificulties in transfer from sitting position to standing position;
  (4) Retaining of cognitive functions and communication skills for good cooperation with the physical therapist and active participation;
  (5) Tolerance to effort;
  (6) First presentation in rehabilitation unit;
  (7) Independent gait without assistive device.
2. Exclusion criteria
  (1) Haemoragic stroke;
  (2) Multiple stroke;
  (3) Other neurologic diseases that affect muscle mass;
  (4) Other diseases such as hepatitis and renal failure;
  (5) Lack of family agreement.

The research was made under the rules of Declaration of Helsinki (2013) and all patients sign informed consent. The research was also approved by the Ethic Commission of the Research Department (University of Craiova-Faculty of Physical Education and Sport).

#### 2.1.2. Evaluation Methods

*Clinical evaluation* refers to evaluation of the side lesion and motor deficiency type (hemiplegia or hemiparesis).

**Figure 1:**
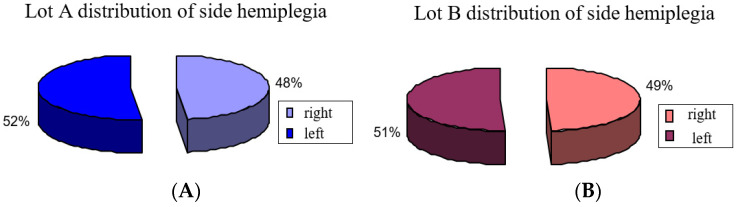
Distribution of side hemiplegia: (**A**) group A, (**B**) group B.

For this we used neurologic variables for groups A and B side lesion and motor deficiency and we observed that it was about the same distribution: group A had 52% of patients with left lesion and 48% with a right lesion, while group B had 51% patients with left side lesion and 49% right lesion (Figure 1).

**Figure 2:**
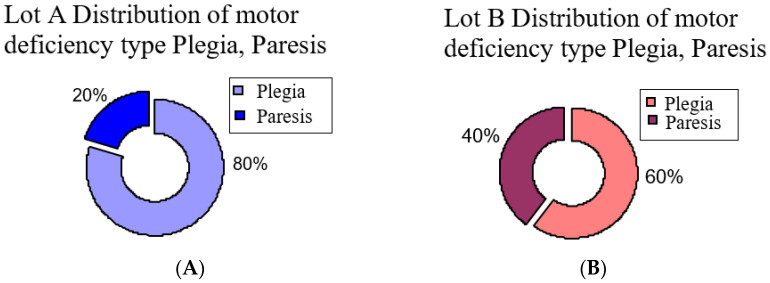
Distribution of motor deficiency type hemiplegia and hemiparesis: (**A**) group A, (**B**) group B.

The second aspect is type of motor deficiency, and we observed in group A (those who started the program in the first month after stroke) a plegic motor deficiency in 80% of patients, and for group B (who started the program later) a plegic motor deficiency in 60% of patients (Figure 2). The rest of the patients, 20% from group A and 40% from group B, had hemiparesis.

Functional evaluation includes use of Tinetti scales for balance and gait. 

#### 2.1.3. Biomechanical Evaluation

Biomechanical evaluation allowed us to evaluate the specific status of drop foot. It is a quantitative evaluation that helps in gait assessment, which is regarding functional lower limb motor performance after stroke.

As stated before, this evaluation was performed on a small number of patients from groups A and B organized into two lots, I (25 patients) and II (25 patients), based on inclusion and exclusion criteria.

For the biomechanic assessment we used Footscan Scientific (Version Footscan 2010, RSSCAN International, Olen, Belgium). The platform makes the measurement at 500 Hz and 2D mode, and records both feet. The platform makes the measurement of pressure distribution during the contact of the feet with the platform [14], with 0.6–0.8 s in a gait cycle. 

This platform gave information about the ground reaction force and plantar pressure during the gait. Force is expressed in N and pressure in N/cm^2^, during a gait cycle for a lower limb. This assessment allows us to estimate the lower limb behavior during gait [15,16].

#### 2.1.4. Biomechanical Parameters

Using the force plate gave us the possibility to study the loading on the foot during gait cycle, based on force vector. This helped us to approach the varus or valgus of the foot. For our study we choice to analyze the following parameters:

1. Maximal pressure for plantar regions: Pmax [N/cm^2^];
2. Contact area, represents the area of contact for each plantar region: CA [cm^2^];
3. Pressure distribution during gait;
4. Center of pressure: COP.

Biomechanical evaluation allowed assessment of the specific status of drop foot.

The platform also gives information about: pressure distribution and force distribution in time for each plantar region, loading, active contact area, foot axis, subtalar angle, foot balance anterior/posterior, and center pressure position.

The measurements of the physiologic gait pattern were performed as follows.

We placed the platform at a distance of 6 m length (for facilitation of the normal gait without restriction). The platform was covered by a synthetic material. The patient was instructed to understand how to perform their gait the way they would in normal conditions, at a comfortable speed, according to their motor possibilities. The patient was not allowed to use any assistive devices.

We made the recording for two gait cycles, for both feet, alternative right/left foot.

For each session of the evaluation we made 3 measurements and we took into consideration the best measurement. We took into consideration the best measurement because the patients needed a period of accommodation to make the test reliable and to be natural in their movement. Sometimes, for example, patients hesitated during the gait on the pressure platform and the footprint was not satisfactory. In addition, the other tests needed 3 measurements. For these tests, the following criteria were considered valid for the measurements:

1. Common pattern of the heel contact;
2. Constant speed.

We studied 3 gait phases (from 8 phases): heel contact, midstance (loading of midfoot), and terminal stance (loading of metatarsian region), the last of which depends on tibiotarsian control.

**Figure 3:**
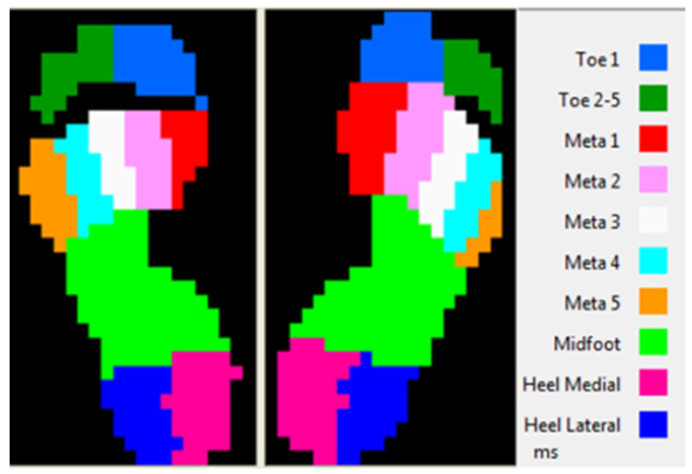
Plantar regions.

Even if the recording were made on the 10 plantar regions (Figure 3), we took in the study only 5 regions:

1. Lateral heel (HL);
2. Medial heel (HM);
3. Midfoot (MF);
4. Toe 1;
5. Toe 5.

We chose these regions because of the importance in gait and anatomic regions of the foot: posterior foot, midlefoot, rarefoot. Furthermore, we made this choice because of the foot position during gait and its response to motor command and control. We recorded measurements for both feet [17].

### 2.2. Statistic Method

Statistic evaluation included the analysis of clinical, functional and biomechanic assessments of gait.

Statistic analysis and graphics were made using general mathematic software (Microsoft Excel) or specific statistics (academic package, MINITAB 15 or OMS package, EPI 2000). The statistical analysis from Microsoft Excel was realized using the predinify functions, mode Data Analysis, XLSTAT and WINSTAT. 

To check the normality of data distribution, we applied the Anderson–Darling test, with significance level 0.05. We applied Kruskal–Wallis test based on numeric values distribution, for analysis of the age distribution for both lots; *ANOVA* test with repeated measurements for both lots; for multiple comparation between average values, we applied *Newman–Keuls* test; Student’s *t*-test was applied for both groups A and B. 

Experimental data were transferred by Windows platform Excel, and we obtain an initial database from which we extracted the significant aspects of this study. By statistical methods we processed the results and we defined some features of the parameters (variables). In addition, by statistical processing we set the significant difference between some of values series and set some correlation between parameters that defined the lots of the patients.

*Therapeutic program* was based on neuromotor facilitation technic (PNF) and functional improvements were based on neuroplasticity proprieties of the central nervous system (training of lower limb and creating a habitat that stimulates neuronal reorganization). The reabilitation program was the same for both lots (lot I and ot II) and included 5 sessions per week, duration 45 min per session.

## 3. Results

### 3.1. Statistic Analysis of the Patient Lots

**Figure 4:**
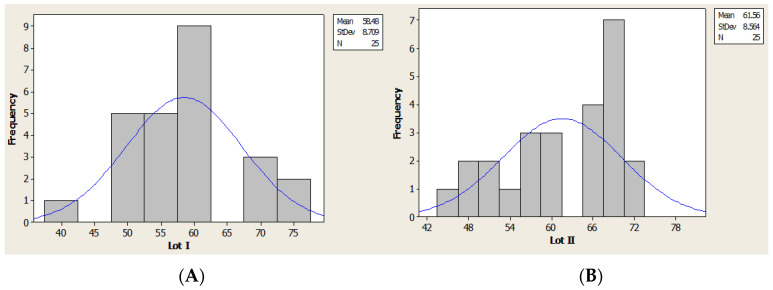
Age distribution (histogram): (**A**) lot I, (**B**) lot II.

Using the Kruskal–Wallis test based on numeric values distribution we made the analysis of age for lot I and lot II (Figure 4).

#### Clinical and Functional Results

For the first moment we included in our study 100 stroke patients: group A (50 patients), group B (50 patients). For both groups we made clinical and functional evaluations. We present in this section the subjects, inclusion and exclusion criteria and also how we made the groups.

**Figure 5:**
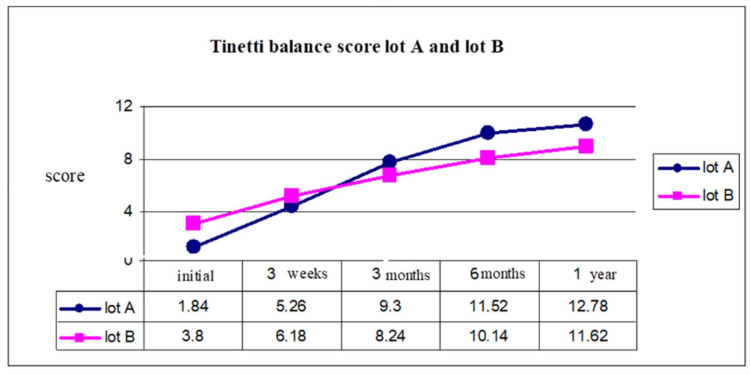
Tinetti balance score group A and group B.

**Figure 6:**
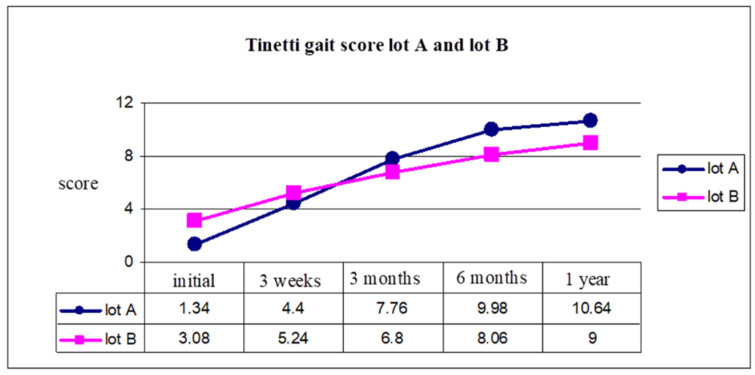
Tinetti gait score group A and group B.

Analysis of the results show us that there was a favorable evolution for group A (who started the rehabilitation program in the first three months after stroke) from a functional point of view. In the next figures we present the evolution of the score of the Tinetti scale for balance(Figure 5) and gait (Figure 6), for five moments of evaluation and medium score after one years from stroke (initial, 3 weeks, 3 months, 6 months, 1 year). 

At the initial moment, the balance evaluation using the Tinetti scale had an average of 1.84 for group A, 3.8 for group B. After one year, because of an early start of the rehabilitation program, we observed that for group A the Tinetti score was 12.78 and for group B was 11.62 (Figure 5).

Gait evaluation using the Tinetti scale for gait showed us a better evolution for group A, with a Tinetti score of 1.34 and 3.08 for group B (initial moment), and 10.64 for group A, and 9 for group B (after 1 year) (Figure 6).

This analysis was made using the Tinetti scale for balance (test 1) and Tinetti scale for gait (test 2). *ANOVA* test with repeated measurements for both lots demonstrated significant differences between group A and group B, with average values of the five moments (Table 1).

Student’s *t*-test was applied to both groups A and B, and the results are presented in Table 2 for initial moment, 3 weeks, 3 months, 6 months and 1 year.

The Student’s *t*-test highlights a significant difference of scores from the initial moment of rehabilitation program and after one year comparing both groups, A (started rehab in the first 3 months) had more significant statistical values than group B (started the program after 3 months). 

### 3.2. Results of Biomechanic Assessment

#### 3.2.1. Distribution of COP in Orthostatic Position

We made the analysis of the COP position according to motor deficiency localization and we observed in left hemiparesis uneven distribution at the initial moment, but good evolution after the rehabilitation program focused on balance training. The result is a recovery of the position at last evaluation.

**Figure 7:**
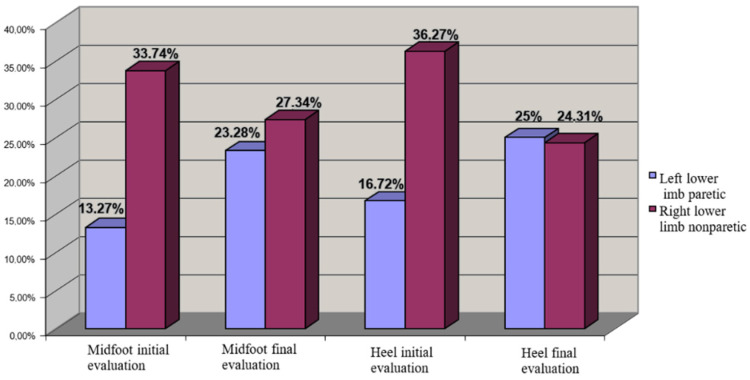
COP distribution for left hemiplegia (left lower limb paretic—cu albastru, right lower limb nonparetic).

In this context we observed that the patients from both lots developed a redistribution of loading from calcaneum to metatarsian V region, and also to the lateral region of plantar side (Figure 7).

**Figure 8:**
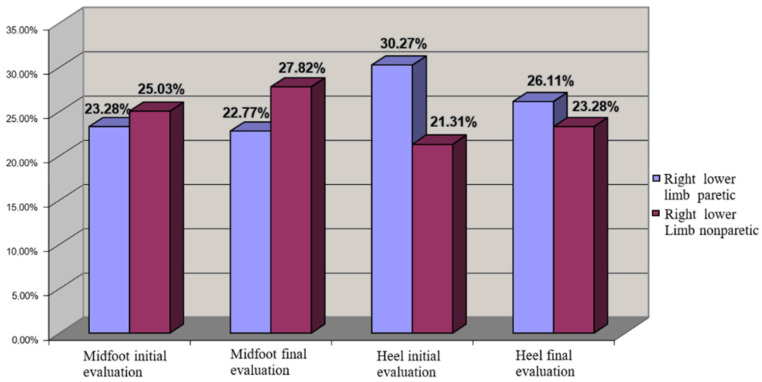
COP distribution for left hemiplegia (left lower limb nonparetic—cu grena, right lower limb paretic).

For right hemiparesis we observed a uniform COP distribution even at the initial moment, which could be explained by motor dominance on the right side, which was a feature of all the patients (from both lots). In this context, any other central imbalance on the left side did not have a major impact on balance. The rehabilitation program improved the distribution of COP and increased the loading on the calcaneum region (Figure 8).

#### 3.2.2. Statistic Analysis of Biomechanical Parameters

As said before, the biomechanic evaluation was performed on patients in lot I and lot II, for contact area (CA) and maximal pressure (Pmax), at 6 months and 1 year after stroke, for all five plantar regions.

#### 3.2.3. Lateral Heel Region

**Figure 9:**
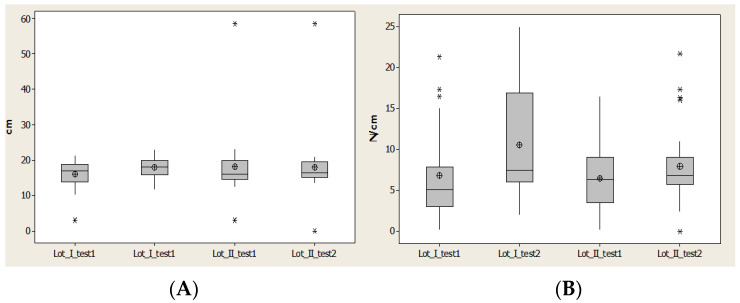
Plantar region lateral heel: (**A**) contact area, (**B**) Pmax distribution. * Small disperssion. ** High distribution.

Contact Area (CA) for lot I after 1 year had the closest values to the average and median values; however this was not the case for lot II. For lot II we observed less distance between quartile 1 and 3 and also for the values interval (Figure 9).

Maximal pressure (Pmax) indicated an increase of the maximal value and alsoincrease for average values and medians for lot I after 1 year. For lot II there was a restriction of the values, but without an increase of average value (Figure 9).

Maximal pressure (Pmax) indicated an increase of maximal value and also increase for average values and medians for lot I after 1 year. For lot II there was a restriction of the values, but without an increase of average value (Figure 9).

**Figure 10:**
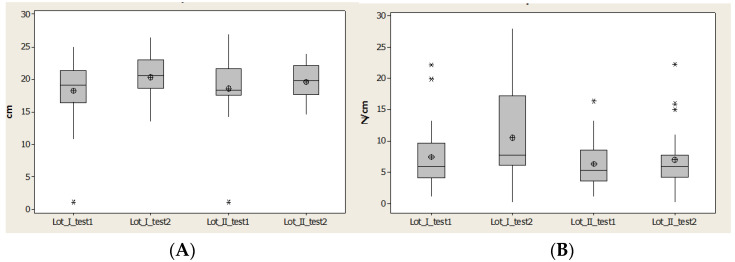
Plantar region medial heel: (**A**) contact area, (**B**) Pmax distribution. * Small distribution, ** High distirbution.

The region medial heel contact area (CA) offered an interval of quartile 1–quartile 3, which is constant for both lots, and the values increased. We observed that there was a restriciton of values for lot II, because of the time between having a stroke and starting rehabilitation (Figure 10). 

Contact area (CA) had an interval of quartile 1–quartile 3, which was constant for both lots and the values increased. We have to notice that it is a restriciton of values for lot II, because of a long time until start the rehabilitation (Figure 10)

Maximal pressure (Pmax) highlights the effect of maximal force due to a relative constant of contact area. We saw very good improvement in lot I patients; the maximal value is three times larger after 1 year than for lot II. The effect of the rehabilitation program, even though it is not homogeneous (some patients have small values), showed us that some patients have high values.

Lot II has three patients out of the normal values and the maximal values decreased, while the average and median values had a moderate increase (Figure 10).

### 3.3. Midfoot Region

**Figure 11:**
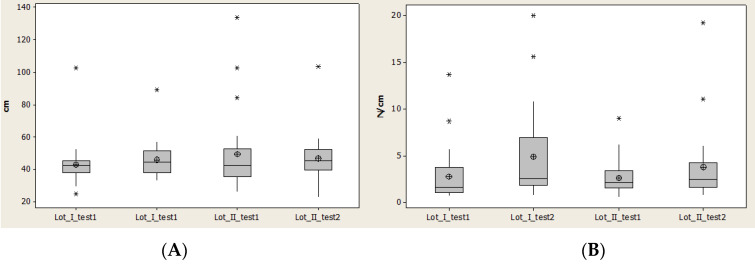
Plantar region midfoot: (**A**) contact area, (**B**) Pmax distribution. * Small distribution, ** High distribution.

Contact area (CA) recorded a decrease of the normal interval and an increase of the interval between quartile 1 and quartile 3 for lot I, and complementary behavior for lot II. Lot II started with high values for CA, which means that a later rehabilitation program did not generate favorable results for this lot (Figure 11).

Maximal pressure (Pmax) had minimal values and also median values relatively constant, and at the same time the average values were different but increased for both lots.

The interval between quartile 1 and 3 also increased, which means that there was not a homogenous distribution of the values.

The high amplitude for lot I demonstrates a faster rehabilitation for lot I than lot II (Figure 11). 

Maximal pressure (Pmax) had minimal values and also median values relatively constant, and at the same time the average values were different but increased for both lots.

The interval between quartile 1 and 3 also increased which means that there was not homogenous distribution of the values.

The high amplitude for lot I demonstrates a faster rehabilitation for lot I than lot II (Figure 11). 

### 3.4. Region Meta 1

Contact area (CA) recorded an increase of normal intervals at second evaluation (after 1 year) for both lots, even though the minimal value for lot I was constant and for lot II decreased.

**Figure 12:**
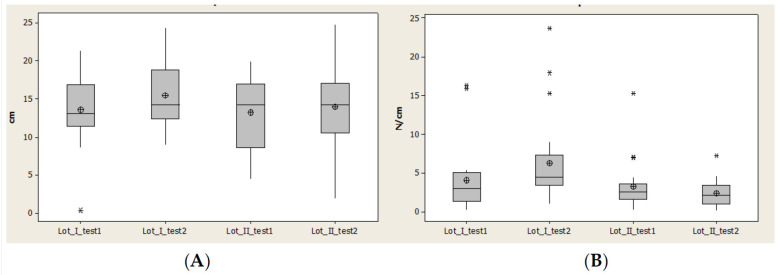
Plantar region midfoot: (**A**) contact area, (**B**) Pmax distribution. * Small distribution, ** High distribution.

In the second evaluation, after 1 year, we observed in lot II a decrease of the interval between quartile 1 and 3 and also a symmetry of data distribution (Figure 12).

Maximal pressure (Pmax) had many values outside of normal interval. In lot I in the second evaluation this number increased and for lot II decreased. The normal interval and interval between quartile 1 and 3 increased for both lots and at the same time the median values and average values decreased for lot II (Figure 12). 

Maximal pressure (Pmax) had many values outside of normal interval. In lot I in the second evaluation this number increased and for lot II decreased. Normal interval and interval between quartile 1 and 3 increased for both lots and at the same time median values and average values decreased for lot II (Figure 12). 

### 3.5. Region Meta 5

**Figure 13:**
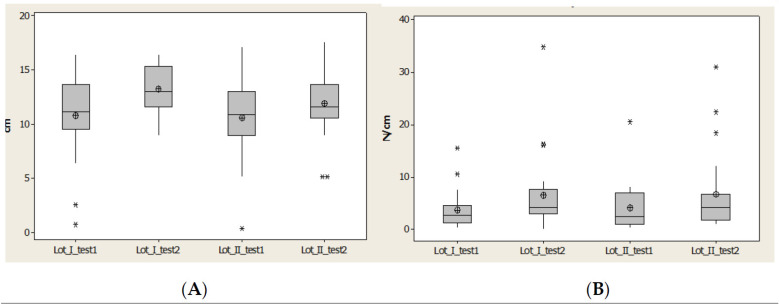
Plantar region Meta 5: (**A**) Contact area, (**B**) Pmax distribution. * Small distribution, ** High distribution.

Contact area (CA) decreased for the normal interval, and the average and median values increased in the second evaluation, after 1 year, for both lots (Figure 13).

Maximal pressure (Pmax) had many values outside of the normal interval. Average and median values between quartile 1 and 3 increased (Figure 13). 

Maximal pressure (Pmax) had many values outside of the normal interval. Average and median values between quartile 1 and 3 increased (Figure 13). 

Evolution of statistics parameters for biomechanic measurements were observed at two moments (6 months and 1 year) for both lots (Table 3, Table 4, Table 5, Table 6 and Table 7).

Statistic analysis of the results revealed that we cannot take into consideration average values because between the two lots there were not significant differences, but we did observe that were significant changes in the studied parameters.

Notable progress was in lot I because of the early rehabilitation program after stroke, which was not the same for lot II. In lot II, the progress WAS almost nonexistent and only incidentally did we observe significant statistical differences.

The interpretation of biomechanic parameters evolution were that CA and Pmax for lot I increased more than 100% (Table 3, Table 4, Table 5, Table 6 and Table 7), which means that there is a favorable evolution of patient health if the patients start the rehabilitation program in the first three months after stroke.

For lot II increases of the parameters were less than lot I (Table 3, Table 4, Table 5, Table 6 and Table 7).

Analysis of the *p*-values show us that there were systematically significant values under 0.05 for lot I and rarely for lot II (Table 8).

## 4. Discussion

The results of the clinical and functional evaluation for group A and B, and for lots I and II (which are from groups A and B) show us that we can speak about a similitude regarding age and genre distribution (homogenous lots and no significant difference).

Regarding the biomechanic parameters, we observed differences regarding CA and Pmax that were recorded during the gait, in correlation with central lesion localization (Figure 7 and Figure 8). Based on these evaluations we saw that the recovery potential of the patients was higher in patients with right hemiparesis, possibly because the muscle damage seemed to be not so severe, which may be a specific morphofunctional feature. This could be explained by the right cerebral dominance of all patients studied, which involves more use of the right side of the body than the left in daily activities. This aspect has also been noticed by other authors [18] in the last years, which speak about the genetic profile of paretic limb compared with the nonparetic limb.

Many studies have observed major differences between paretic and nonparetic lower lim, from the genetic expression point of view. This is an aspect also demonstrated by this research, based on clinical, functional and biomechanic evaluation.

This specific genetic expression is visible on muscle metabolism, muscle contraction proprieties, rate of cells multiplication, growth factor and mitogenesis.

The most affected gene is the gene that controls the muscle metabolism, therefore the oxidative enzymes are influenced by genetic changes and impact the muscles of the paretic limb.

These changes are in corellation with the MHC isoform, indicating that we observed an anaerobic metabolism of paretic limbs [19] and an increase of fast muscle fibers.

The presence of muscle fibers in high percentage explains the lack of coordination and motor control which can be highlighted by the position of COP, Pmax and CA.

All of these are very important if corellated with kinetic parameters, and can help to design a specific rehabilitation program based on muscle plasticity. This program could be more efficient because is based on measurements and not just clinical aspects which do not allow time to adapt the rehabilitation program.

When analyzing the functional evaluation, we can posit that in the first six months there were not great increases in the values, perhaps because the rehabilitation program does not take into consideration specific muscle structure changes. This evolution was similar for both lots.

The data presented in Table 3, Table 4, Table 5, Table 6 and Table 7 show us that kinematic parameters for plantar regions have the following evolution:

- Lateral heel: significant increase of CA.
- Medial heel: in lot I was significant increase of CA and for lot II a decrease.
- Midfoot: greater increase of the CA for lot I than lot II, which means significant motor deficiency in lot II, due to spasticity of triceps sural.
- Metatarsian I (Meta I): for lot I all parameters had an significant increase, which means a good evolution and a good response of this region under the rehabilitation program, due to a physiological position of the foot and reduction of the inversion and plantar flexion. For lot II we observed a decrease of the parameters (CA and Pmax).
- Metatarsian V (Meta V): increased in all parameters and this is corellated with the evolution of Meta I. This suggests that the longitudinal axis of the foot had a physiological loading for lot I, but at the same time for lot II we observed a decrease of CA and Pmax.

Starting from gait phases and because the plantar movement is from posterior to anterior side, by heel attack, and the lateral heel of the plantar region and loading of the midfoot and metatarsian region, we can observe that in lot I there was an important improvement of biomechanic parameters and an improvement of heel position on the ground. For lot II the loading decreased with time, meaning that the foot did not restore its balance and the motor control was not further developed due to the later rehabilitation program start and loss of cerebral engrams. This aspect was also studied by other authors [20] using the biomechanic analysis of gait. However, this was not the same for lot I, which had an improvement of CA and Pmax for the medial heel region at the first moment, but a decrease at the second evaluation with an increase on the lateral side of plantar region loading. This could be considered an evolution to a normal position of the foot.

The midfoot also recorded also an increase of biomechanic parameters for lot I which were in accordance with evolution for Meta I and Meta V. This indicates a uniform distribution of plantar loading during gait in lot I. For lot II we observed a decrease of CA because of a loss of the functional skills of the foot and morphofunctional damage of the paretic limb. The result decreases the chance for gait rehabilitation. Analysis of the biomechanic aspect reveals a lot of diversity regarding compensatory mechanisms that are developed for the paretic and nonparetic lower limb. For this reason a lot of authors report that motor control is a problem in motor recovery of stroke patients. More so on the paretic limb, but also on the nonparetic limb, are many compensatory mechanisms based on damage the sensorio motor aspects that could generate change of gait strategy [11].

Development of different movement patterns due to muscle force changes also include a selective loss of the muscle fibers in type II and increase the fibers type I [1].

This selective loss of fibers in type II generates disorders of fast movement and force. The number of motor units (MU) decreases more than 50% after 6 months, and this is the result of damage to the synaptic transmission on the alpha motoneuron due to loss of cortico-spinal transmission [2].

Biomechanic evaluation aspects revealed that after strokes there are a lot of adaptive phenomena which require strategies. These strategies can be found also in healthy people during different motor actions, such as gait in irregular terrain [21]. Formisano et.al [22] highlighted the importance of an early start of rehabilitation programs regarding the complexities of rehab after stroke. They studied patients with flaccid paralysis and observed that patients have a plateau phase of muscle tone at three months after stroke. The patients with spasticity develop the spasticity in less than three months; this is one more argument supporting the importance of starting the rehabilitation program early [22].

The results of our study are also in accordance with Hsiao et al. [23], who observed that the weight transfer of stroke patients has a lot of abnormal aspects regarding control of the transfer due to the decrease of knee flexion and ankle, damage to the interjoint play, and reduction of the chance for having stability of COP. This means a difficulty to regain neuromuscular control.

For this reason, the authors consider that more information is needed about biomechanic evaluation parameters that could reveal the status of the proprioceptive system, which can help the development of a focused rehabilitation program. This is an accordance with our results and aim of our research.

Aspects regarding localization of cerebral damage, right or left, are also a challange for prediction of patient evolution after stroke, and are also presented by Frenkel et. al in their study [24]. They studied the relationship between localization of cerebral damage and evolution of gait in the long term and concluded that there are a lot of variables regarding neuronal network reorganization in a closed relationship with cerebral dominance. In addition, they emphasized the importance of others’ studies.

The importance of biomechanic plantar parameters, -mainly COP, is also presented by Gray [25] in his study about the requirement of monitoring the effects of Constraint-Induced Movement (CIM) by analysis of the COP toward the affected limb [25].

The results of our research demonstrate that the optimal time for starting the rehabilitation program is before three months after stroke. Comparing our results with other similar studies, we can see that there are significant statistic differences betweem lot I and lot II, in the sense of faster recovery in lot I than in lot II. This aspect is also presented by Gray [25] and Bernhardt [26].

Evaluation of patients in the Bernhardt study were made using a single scale, and this allows us to conclude that a complex evaluation that also includes biomechanic evaluation gives more information about the results of a rehabilitation program in the first three months after stroke. Meta-analysis made in the study of Diserens et al. [27] demonstrated also that there is not enough information about the efficency of early mobilization; however in our study we demonstrated that this is a real approach, proven by the complex clinical functional and biomechanic evaluation. 

**Limitation of the study**: The sample size in this study was not very large, therefore new research on a larger sample could allow extending the measurements and finding more significant relationships from the data.

Furthermore, there only exist a small number of studies in the literature regarding such analysis that we proposed in this research, therefore we cannot compare the data.

Data collection could be affected by the patients’ cooperation. Some patients had hesitation while walking on the platform even if the procedure was explained.

Participation in the rehabilitation program sometimes has problems because not all patients attended every session each week.

## 5. Conclusions

Biomechanic evaluation of CA and Pmax in the lateral heel and medial heel is relevance for starting the rehabilitation program and confirming the good evolution for the people that start the rehabilation before three months after stroke. Localization of cerebral lesion, even from a statistical point of view, did not present significant differences, still biomechanic evaluation demonstrated a good evolution for right hemiplegia. We observed that in the rehabilitation program for gait, kinematic variables have descriptive values in practical activity, and kinetic variables have explanatory value and help to understand movement. They are also important for designing the rehabilitation program and evaluating its results.

We consider that this study is useful for monitoring patient rehabilitation programs so that rehabilitation after stroke could be more efficient.

## Figures and Tables

**Table 1:** *ANOVA* test 1 and test 2, for group A and B.

Lot	Test	F	*p*	Statistic Significance
group A	1	1016.95	<0.001	Yes
	2	986.08	<0.001	Yes
group B	1	660.45	<0.001	Yes
	2	518.69	<0.001	Yes

**Table 2:** Student’s *t*-test test for group A and B, for each testing.

Test	Test 1 Initial Test		Test 2 3 Weeks		Test 3 3 Months		Test 4 6 Months		Test 5 1 Year		Statistic Significance
	*t*	*p*	*t*	*p*	*t*	*p*	*t*	*p*	*t*	*p*	
1	−5	<0.001	−1.91 *	0.325 *	2.32 ***	0.022 ***	3.11	0.002	3.36	0.001	Yes
2	−5.53	<0.001	−2.46 **	0.016 **	2.81	0.006	5.45	<0.001	7.08	<0.001	Yes

* indicates no significant differences between average values for two groups (|t_calculate_| < t_table_ and *p* > 0.05). ** indicates significant differences between average values for two groups (|t_calculate_| > t_table_ and *p* < 0.02). *** indicates significant differences between average values for two groups (|t_calculate_| > t_table_ and *p* < 0.05), without * indicates significant differences between average values for two groups (|t_calculate_| > t_table_ and *p* < 0.01), if *t* is negative, then the average for group B is more than average for group A, **t_table_** = 2.575 significant level 1%; **t_table_** = 2.326 significant level 2%; **t_table_** = 1.959 significant level 5%.

**Table 3:** Average values for lateral heel, 6 months and 1 year.

Lateral Heel				
T	Lot	Statistic Parameter	Contact Area (cm^2^)	Pmax (N/mm^2^)
**T1 6 months**	**Lot I**	**average**	15.95	6.80
		dev.std.	4.2508	5.4714
	**Lot II**	**average**	17.49	6.24
		dev.std.	9.9963	3.8978
	**Lot I/II**	***p***	0.4874	0.6895
**T2 1 year**	**Lot I**	**average**	17.90	10.56
		dev.std.	3.1388	7.3425
	**Lot II**	**average**	17.97	7.99
		dev.std.	9.3480	5.0262
	**Lot I/II**	***p***	0.9727	0.1563

**Table 4:** Average values for medial heel, 6 months and 1 year.

Medial Heel				
T	Lot	Statistic Parameter	Contact Area (cm^2^)	Pmax (N/mm^2^)
**T1 6 months**	**Lot I**	**average**	18.31	7.48
		dev.std.	5.1069	5.3245
	**Lot II**	**average**	18.60	6.43
		dev.std.	4.7670	3.8229
	**Lot I/II**	***p***	0.8454	0.4422
**T2 1 year**	**Lot I**	**average**	20.40	10.56
		dev.std.	3.2666	7.3128
	**Lot II**	**average**	19.67	7.05
		dev.std.	2.6819	4.9113
	**Lot I/II**	***p***	0.3967	0.0540

**Table 5:** Average values for midfoot, 6 months and 1 year.

Midfoot				
T	Lot	Statistic Parameter	Contact Area (cm^2^)	Pmax (N/mm^2^)
**T1 6 months**	**Lot I**	**average**	43.10	2.83
		dev.std.	14.1564	3.0215
	**Lot II**	**average**	49.45	2.68
		dev.std.	24.3512	1.9230
	**Lot I/II**	***p***	0.2691	0.8472
**T2 1 year**	**Lot I**	**average**	45.85	4.88
		dev.std.	11.2718	4.8778
	**Lot II**	**average**	46.79	3.83
		dev.std.	14.0538	3.9213
	**Lot I/II**	***p***	0.7953	0.4067

**Table 6:** Average values Meta 1, 6 months and 1 year.

Meta 1				
T	Lot	Statistic Parameter	Contact Area (cm^2^)	Pmax (N/mm^2^)
**T1 6 months**	**Lot I**	**average**	13.64	4.10
		dev.std.	4.5151	4.4424
	**Lot II**	**average**	13.24	3.28
		dev.std.	4.7089	3.1724
	**Lot I/II**	***p***	0.7756	0.4984
**T2 1 year**	**Lot I**	**average**	15.52	6.30
		dev.std.	4.3067	5.5708
	**Lot II**	**media**	13.97	2.39
		dev.std.	5.0318	1.6515
	**Lot I/II**	***p***	0.2630	0.0033 *

* *p* ≤ 0.01.

**Table 7:** Average values Meta 5, 6 months and 1 year.

Meta 5				
T	Lot	Statistic Parameter	Contact Area (cm^2^)	Pmax (N/mm^2^)
**T1 6 months**	**Lot I**	**average**	10.80	3.79
		dev.std.	3.8836	3.7366
	**Lot II**	**average**	10.64	4.13
		dev.std.	3.5676	4.5148
	**Lot I/II**	***p***	0.8902	0.7889
**T2 1 year**	**Lot I**	**average**	13.31	6.63
		dev.std.	2.1230	7.1486
	**Lot II**	**average**	11.93	6.78
		dev.std.	3.2544	7.7071
	**Lot I/II**	***p***	0.0984	0.9456

**Table 8:** Statistic significance of biomechanic parameters evolution of two lots, at 6 months and 1 year, to the plantar regions.

	Values *p* Kruskal–Wallis Test Test 1/Test 2		
Plantar Region	Lot	Contact Area	Pmax
**Lateral heel**	**Lot I**	0.107	0.014
	**Lot II**	0.834	0.441
**Medial heel**	**Lot I**	0.066	0.066
	**Lot II**	0.386	0.845
**Midfoot**	**Lot I**	0.187	0.02
	**Lot II**	0.522	0.337
**Meta 1**	**Lot I**	0.209	0.046
	**Lot II**	0.718	0.483
**Meta 5**	**Lot I**	0.013	0.027
	**Lot II**	0.208	0.153

## References

[1] Ramsay J.W., Barrance P.J., Buchanan T.S., Higginson J.S. (2011). Paretic muscle atrophy and non-contractile tissue content in individual muscles of the post-stroke lower extremity. J. Biomech..

[2] Prado-Medeiros C.L., Silva M.P., Lessi G.C. (2012). Muscle atrophy and functional deficits of knee extensors and flexors in people with chronic stroke. Phys. Ther..

[3] Batchelor F.A., Mackintosh S.F., Said C.M., Hill K.D. (2012). Falls after stroke. Int. J. Stroke.

[4] Mackintosh S.F., Goldie P., Hill K. (2005). Falls incidence and factors associated with falling in older, community—Dwelling, chronic stroke survivors (>1 year after stroke) and matched controls. Aging Clin. Exp. Res..

[5] Criciotoiu A.O., Stanca D.I., Bondari S., Malin R.D., Ciolofan M.S., Schenker M. (2019). Correlation between the Age, Motor Subtypes and the Necessity of Advanced Therapy in Parkinson Disease. Rev. Chim..

[6] Pinter M.M., Brainin M. (2012). Rehabilitation after stroke in older people. Maturitas.

[7] Weerdesteyn V., De Niet M., van Duijnhoven H.J., Geurts A.C. (2008). Falls in individuals with stroke. J. Rehabil. Res. Dev..

[8] Caliskan Uckun A., Celik C., Ucan H., Ordu Gokkaya N.K. (2014). Comparison of effects of lower extremity orthoses on energy expenditure in patients with cerebral palsy. Dev. Neurorehabilit..

[9] Richards A., Morcos S., Rethlefsen S., Ryan D. (2012). The use of TheraTogs versus twister cables in the treatment of in-toeing during gait in a child with spina bifida. Pediatric Phys. Ther..

[10] Becerro-de-Bengoa-Vallejo R., Losa-Iglesias M.E., Rodriguez-Sanz D. (2014). Static and dynamic plantar pressures in children with and without sever disease: A case-control study. Phys. Ther..

[11] Winter D.A. (1995). Human balance and posture control during standing and walking. Gait Posture.

[12] Fuller E.A. (1999). Center of pressure and its theoretical relationship to foot pathology. J. Am. Podiatr. Med. Assoc..

[13] Criciotoiu A.O., Stanca D.I., Glavan D.G., Bondari S., Malin R.D., Ciolofan M.S. (2019). The Relations between Non-motor Symptoms and Motor Symptoms in Parkinson Disease. Rev. Chim..

[14] Tavares J.M.R.S., Oliveira F.P.M. (2014). Novel framework for registration of pedobarographic image data. Med. Biol. Eng. Comput..

[15] Keijsers N.L.W., Stolwijk N.M., Pataky T.C. (2010). Linear dependence of peak, mean, and pressure—Time integral values in plantar pressure images. Gait Posture.

[16] Low D.C., Dixon S.J. (2010). Footscan pressure insoles: Accuracy and reliability of force and pressure measurements in running. Gait Posture.

[17] Libardoni dos Santos J.O., Manfio E.F., Carpes F.P., Bezerra E.S., Palhano R., Avila A.O.V. (2017). Change of Pronation Angle of the Subtalar Joint has Inluence on Plantar Pressure Distribution. Rev. Bras. Cineantropometria Desempenho Hum..

[18] Ivey F.M., Ryan A.S., Hafer-Macko C.E., Garrity B.M., Sorkin J.D., Goldberg A.P., Macko R.F. (2006). High prevalence of abnormal glucose metabolism and poor sensitivity of fasting plasma glucose in the chronic phase of stroke. Cerebrovasc. Dis..

[19] Tarnopolsky M.A. (2004). Body-weight-support treadmill training improves blood glucose regulation in persons with incomplete spinal cord injury. J. Appl. Physiol..

[20] Chen C., Patten C., Kothari D.H. (2005). Gait differences between individuals with post-stroke hemiparesis and non-disabled controls at matched speeds. Gait Posture.

[21] Panizzolo F., Lee S., Miyatake T., Rossi D.M., Siviy C., Speeckaert J. (2017). Lower limb biomechanical analysis during an unanticipated step on a bump reveals specific adaptations of walking on uneven terrains. J. Exp. Biol..

[22] Formisano R., Pantano P., Buzzi M.G., Vinicola V., Penta F., Barbanti P., Lenzi G.L. (2005). Late motor recovery is influenced by muscle tone changes after stroke. Arch. Phys. Med. Rehabil..

[23] Hsiao H.Y., Gray V.L., Borrelli J. (2020). Biomechanical control of paretic lower limb during imposed weight transfer in individuals post-stroke. J. Neuroeng. Rehabil..

[24] Frenkel-Toledo S., Ofir-Geva S., Mansano L., Granot O., Soroker N. (2021). Stroke Lesion Impact on Lower Limb Function. Front. Hum. Neurosci..

[25] Gray C.K., Culham E. (2014). Sit-to-Stand in People with Stroke: Effect of Lower Limb Constraint-Induced Movement Strategies. Stroke Res. Treat..

[26] Bernhardt J. (2008). Very early mobilization following acute stroke: Controversies, the unknowns, and a way forward. Ann. Indian Acad. Neurol..

[27] Diserens K., Patrik M., Julien B. (2006). Early mobilization after stroke. Cerebrovasc. Dis..

